# Research on predicting 2D-HP protein folding using reinforcement learning with full state space

**DOI:** 10.1186/s12859-019-3259-6

**Published:** 2019-12-24

**Authors:** Hongjie Wu, Ru Yang, Qiming Fu, Jianping Chen, Weizhong Lu, Haiou Li

**Affiliations:** 1 School of Electronic and Information Engineering, Suzhou University of Science and Technology, Suzhou, 215009 China; 2 Jiangsu Province Key Laboratory of Intelligent Building Energy Efficiency, Suzhou University of Science and Technology, Suzhou, 215009 China

**Keywords:** Reinforcement learning, HP model, Structure prediction

## Abstract

**Background:**

Protein structure prediction has always been an important issue in bioinformatics. Prediction of the two-dimensional structure of proteins based on the hydrophobic polarity model is a typical non-deterministic polynomial hard problem. Currently reported hydrophobic polarity model optimization methods, greedy method, brute-force method, and genetic algorithm usually cannot converge robustly to the lowest energy conformations. Reinforcement learning with the advantages of continuous Markov optimal decision-making and maximizing global cumulative return is especially suitable for solving global optimization problems of biological sequences.

**Results:**

In this study, we proposed a novel hydrophobic polarity model optimization method derived from reinforcement learning which structured the full state space, and designed an energy-based reward function and a rigid overlap detection rule. To validate the performance, sixteen sequences were selected from the classical data set. The results indicated that reinforcement learning with full states successfully converged to the lowest energy conformations against all sequences, while the reinforcement learning with partial states folded 50% sequences to the lowest energy conformations. Reinforcement learning with full states hits the lowest energy on an average 5 times, which is 40 and 100% higher than the three and zero hit by the greedy algorithm and reinforcement learning with partial states respectively in the last 100 episodes.

**Conclusions:**

Our results indicate that reinforcement learning with full states is a powerful method for predicting two-dimensional hydrophobic-polarity protein structure. It has obvious competitive advantages compared with greedy algorithm and reinforcement learning with partial states.

## Background

The biological function of proteins is determined by their spatial folding structure. Understanding the folding process of proteins is one of the most challenging issues in the field of bioinformatics [1]. The bioinformatics hypothesis believes that the protein form found in nature is the most stable form (the lowest free energy). Protein sequences determine protein structure, and protein structure determines protein function [2, 3]. Current research has put forward many computational theoretical models, such as hydrophobic polarity (HP) model, AB off-lattice model (Toy model) and continuous model of Euclidean space. The HP model is a widely studied simplified protein folding model with high confidence in the prediction of the protein helical structure. In this model, each amino acid is treated either hydrophobic (H) or hydrophilic (P) and represented as a point on a two-dimensional lattice structure. The rationale behind the HP model is that the hydrophobicity of amino acids is the main driving force for small globulins to form a natural conformation [4]. The primary structure analysis of protein sequences involves the analysis of amino acid physicochemical properties (such as hydrophilicity and hydrophobicity) and sequence patterns, so 2D-HP protein structure prediction refers to predicting the folding structure based on the primary structural analysis of proteins. Although the HP grid model is a simplified model, solving the protein folding problem of this model is still difficult. This problem has proven to be an NP-hard problem, which means that there is no solution algorithm that is both complete and not too slow.

Currently, the methods used to solve the HP model optimization include evolutionary algorithm (EA), genetic algorithm (GA), ant colony optimization (ACO), and supervised classification methods. Genetic algorithm is a method of searching for optimal solutions by simulating natural evolutionary processes. The asymptotic analysis of the computational complexity of the GA and EA is difficult and is usually limited to specific problems [5, 6]. The ACO is a probabilistic algorithm used to find optimized paths. In most cases, the ACO has high computational complexity [7–9]. Supervised classification is a method of pattern recognition, and its training process requires external supervision [10–12]. The versatility of these methods is not good, especially when calculating the energy minimum, it is easy to fall into the local optimal solution, which makes it difficult to achieve global optimization [13–15]. Protein structure prediction has two major problems. The first question is how to abstract the mathematical model that can reflect the interaction between amino acids and how to design its energy function. The second problem is how to find an efficient search method for the exploration of the structure and then find the structure with the lowest energy [16–18] within limited central processing unit power and time.

Recently, reinforcement learning has been successfully applied to many aspects of the biological field, such as biological sequence comparisons, genome sequencing and so on, and it has become more extensive in other fields, such as vehicle positioning and recognition, game automation detection and robot simulation [19]. The advantage of reinforcement learning is that the training process does not require external supervision. The agent will conduct autonomous learning based on their interaction experience with the environment, and can find the overall optimal solution based on the reward, and it is not easy to fall into the local optimum [20]. For example, transfer learning in reinforcement learning is considered to be an optimal learning strategy under limited data conditions, especially in areas where labeling data are scarce and distribution is heterogeneous, such as clinical medical diagnosis and animal behavior control [21].

Therefore, this paper proposes an HP model optimization method based on reinforcement learning. In the reinforcement learning framework, the state set and state transition space are given according to the length of the HP sequence to be tested. The agent uses the Q-learning algorithm to select different actions under different conditions to obtain different reward values, and continuously calculates and updates the Q-value table. At last, the agent selects the optimal solution to obtain the optimal structure according to the converged Q-value table. This method has strong universality and simple calculation [22]. It can predict the optimal structure well for short length sequences.

## Methods

### The framework based on reinforcement learning

In recent years, some scholars have proposed some simplified models for protein folding problems. The most typical one is the two-dimensional hydrophobic-polarity (2D-HP) grid model proposed by Dill et al. [23]. According to the differences in the hydrophilicity and hydrophobicity of each type of amino acid, they are divided into two categories: one is a hydrophobic amino acid (indicated by H, the black circle), and the other is a hydrophilic amino acid (indicated by P, the white circle), so any protein chain can be expressed as a finite-length string of H and P [24, 25]. A legitimate protein space configuration must meet the following three constraints:

- ① The center of the sphere for each circle in the sequence must be placed on an integer coordinate in two dimensions.
- ② Any two adjacent circles in the chain must be adjacent to each other in 2D space. That is, the distance between adjacent numbered circles is 1.
- ③ Each integer grid in 2D space can only represent one circle at most, that is, no two balls overlap.

**Fig. 1:**
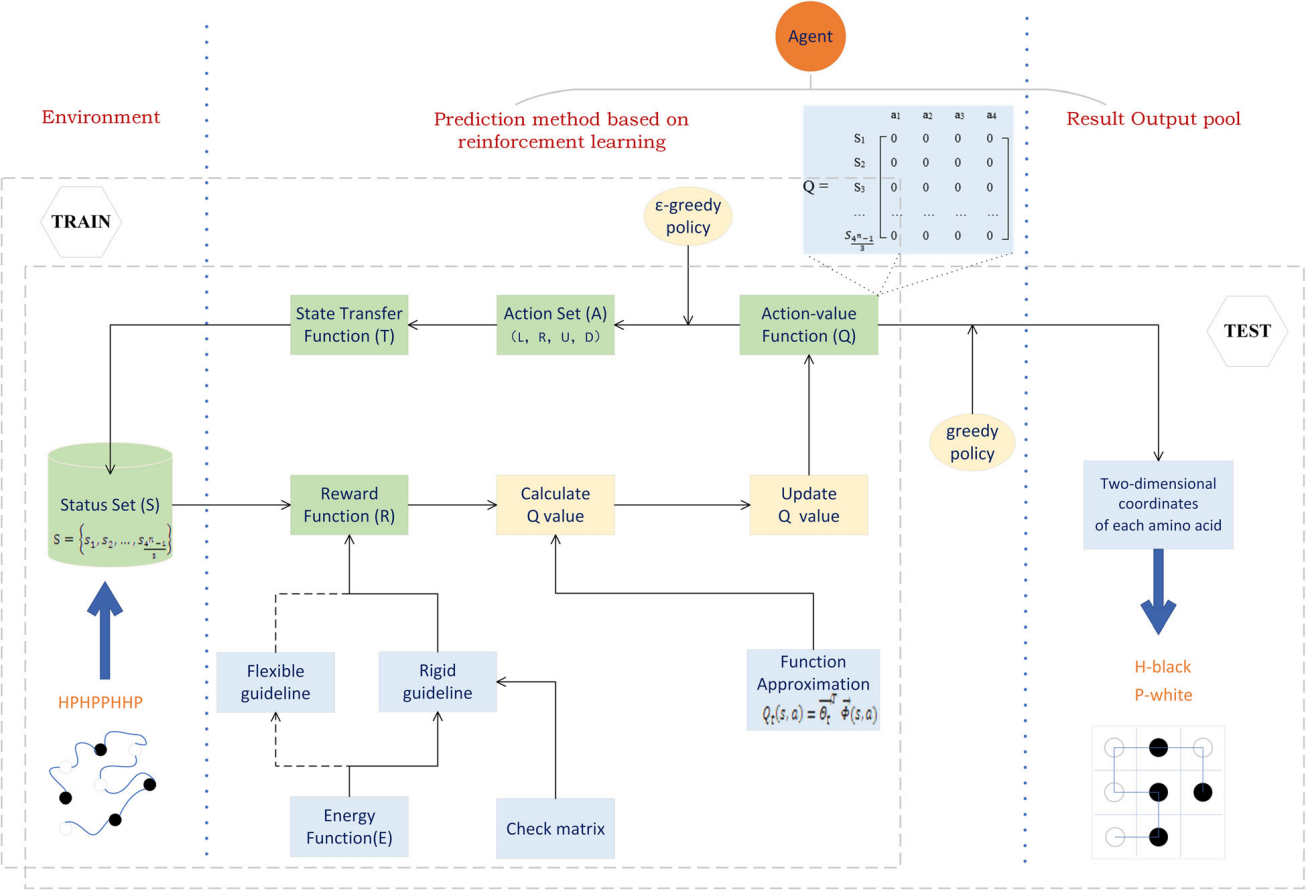
A framework for 2D-HP protein folding based on reinforcement learning with full states

The reinforcement learning method is used to solve the HP 2D sequence model optimization problem, which can be converted into a Markov decision process and solved by the Q-learning algorithm. The framework is shown in Fig. 1.

#### Environment

Amino acids are classified into H (hydrophobic) and P (hydrophilic) according to their hydrophilicity and hydrophobicity. In this case, the amino acid sequence is converted into an HP sequence. Using the HP sequence as input data, the entire state set *S* of the sequence corresponds to the environment part of reinforcement learning.

#### Action set A

Action set *A* consists of 4 actions that corresponds to four directions: L (Left), U (Up), R (Right), D (Down), that is *A* = {*a*_1_, *a*_2_, *a*_3_, *a*_4_}, where *a*_1_ = L, *a*_2_ = U, *a*_3_ = R, *a*_4_ = D.

In the training process, the agent uses ε-greedy policy to select the action (***ε*** ∈ [0, 1]), which means that the agent will explore other actions with the probability of ε and the probability of remaining 1 − ***ε*** goes “greedy “, which means that the agent takes the best action, and constantly calculates and updates the *Q* value [26].

#### Result output pool

The theory shows that, as long as the number of training is enough, the *Q* value will converge to the optimal value. At the end of training, the agent adopts greedy policy to choose the optimal action in different states according to the converged *Q* value to further obtain the optimal structure of HP model. Different folded structures with the lowest energy are the final output results.

### The full state set S of 2D-HP model

The initial state of the agent in the environment is *s*_1_. For a two-dimensional sequence of length *n*, its state space *S* consists of $\frac{4^n-1}{3}$ states. When the state of the first amino acid is fixed, all possible states of the successor of each amino acid are the collection of four states (up, down, left, right) of the previous amino acid, that is, the number of all possible states of subsequent amino acid is four times the number of previous amino acids. The total number of the state set is the sum of the geometric series with an initial value of 1 and an odds ratio of 4, as shown in Eq. (1):

1 $$ S=\frac{1\times \left(1-{4}^n\right)}{1-4}=\frac{4^n-1}{3} $$

So $S=\left\{{s}_1,{s}_2,\dots, {s}_{\frac{4^n-1}{3}}\right\}$. For example, when there is only one amino acid in the sequence, there is only one state *s*_1_ in the whole state space. When there are two amino acids, the possible state of the second amino acid consists of four states of the first amino acid *s*_2_, *s*_3_, *s*_4_, *s*_5_, so there are 5 states *s*_1_, *s*_2_, *s*_3_, *s*_4_, *s*_5_ in the whole space. Similarly, when there are three amino acids, the possible states of the third amino acid consist of four states (up, down, left, right) of the second amino acid, and the second amino acid may have four states, so the third amino acid may have 16 states, namely *s*_6_, *s*_7_ … *s*_20_, *s*_21_, and the whole state set has 21 states, and so on, all the states of subsequent amino acids are obtained.

At the same time, we need to define the state transfer function *T* : *s* → *s*^′^ of the HP model, that is, *T*(*s*, *a*) = *s*^′^*.* The process that the agent takes the action *a* in the state *s* to the subsequent state *s*^′^ can be written as the concrete expression as shown in Eq. (2)

2 $$ T\left({s}_{\frac{4^{i-1}-1}{3}+k},{a}_l\right)={s}_{\frac{4^i-1}{3}+4\times \left(k-1\right)+l} $$

where, *i* ∈ [1, *n* − 1] is the index of the amino acid in the sequence. *k* ∈ [1, 4^*i* − 1^] represents the *kth* state of all the states of the *i-1th* amino acid. *l* ∈ [1, 4] represents the number corresponding to the action.

This means that the agent can move to one of four possible successor states from the state *s*∈ *S* by performing one of four possible actions. It should be noted that each state *s*^′^ ∈ *S* can be accessed from the state *s.*

### The new definition of full state space of 2D-HP model

Further research has found that when the number of actions is reduced to three, a simpler representation of the state space can be obtained. Action set can be described as *A* = {*a*_1_, *a*_2_, *a*_3_}, where *a*_1_ = Left, *a*_2_ = Up, *a*_3_ = Right. Then for a two-dimensional sequence of length *n*, the state space *S* has $\frac{3^n-1}{2}$ states. The number of states is calculated in the same as before, as shown in Eq. (3):

3 $$ S=\frac{1\times \left(1-{3}^n\right)}{1-3}=\frac{3^n-1}{2} $$

So $S=\left\{{s}_1,{s}_2,\dots, {s}_{\frac{3^n-1}{2}}\right\}$. Accordingly, the state transfer function is updated to Eq. (4):

4 $$ T\left({s}_{\frac{3^{i-1}-1}{2}+k},{a}_l\right)={s}_{\frac{3^i-1}{2}+3\times \left(k-1\right)+l} $$

where, *i* ∈ [1, *n* − 1] is the index of the amino acid in the sequence. *k* ∈ [1, 3^*i* − 1^] represents the *kth* state of all the states of the *i-1th* amino acid. *l* ∈ [1, 3] represents the number corresponding to the action.

### Energy-based reward function with criterions

The protein folding thermodynamic hypothesis holds that the energy of proteins under natural structures is the lowest [27, 28]. Therefore, the problem of predicting protein folding structures is to find the lowest energy structure of all available structures for a given amino acid sequence. The determination of the energy function is especially important for this paper.

The energy value is only determined by the hydrophobic force. Each pair of hydrophobic amino acids that is not adjacent in sequence but adjacent in 2D space produces energy of − 1, and in other cases, the energy is calculated as 0. The energy value of the entire structure is the sum of energy of each pair of hydrophobic amino acids that meets the requirements mentioned above in the legal configuration. A formal description of the legal configuration energy *E* of a protein of chain length *n* is as follows:

5 $$ E={\sum}_i^{n-1}{\sum}_{j=i+1}^n{W}_{ij} $$

where, *n* is the length of the amino acid sequence. Both *i* and *j* are the indices of the amino acids in the sequence. And

6 $$ {W}_{ij}=\left\{\begin{array}{c}-1, applicable\ conditions\\ {}0, other\ cases\end{array}\right. $$

where, applicable conditions mean that the *ith* and *jth* amino acid are both hydrophobic amino acids and they are not adjacent in the sequence but adjacent in 2D space.

The purpose of reinforcement learning is to maximize the objective function, which is to maximize the reward. However, in the HP model problem, the ultimate goal is to minimize the energy function, so we need to take the absolute value of the energy function to achieve the positive unite. At the same time, using the absolute value of the energy function as a reward after reaching the end state enables the trained structure closer to the ideal structure.

In the training process, the agent tends to put amino acids in the lattice which placed in the amino acid before, which is not allowed in the actual situation. This overlapping problem can be solved by setting the reward function. We define the reward function by flexible and rigid criteria.

#### Flexible criterion

- ① When the agent selects the action, they are allowed to place the succeeding amino acid in the lattice position where the amino acid was placed before. A negative reward (which can be defined as a penalty) is given to the agent to judge and optimize to maximize the prize. Before reaching the terminal state, the next state of the amino acid is placed in the invalid position with the reward set to − 10.
- ② Before reaching the terminal state, the next state of the amino acid is placed in the valid position (blank position) with the reward set to 0.
- ③ When the terminal state is reached, the absolute value of the energy of the final folded structure is rewarded.

7 $$ \mathrm{That}\ \mathrm{is}\ R=\left\{\begin{array}{cc}-10,& \begin{array}{c}i\in \left(1\sim n-1\right)\\ {} the\  ith\  amino\ acid\ is\ in\ the\ invalid\ position\end{array}\\ {}0,& \begin{array}{c}i\in \left(1\sim n-1\right)\\ {} the\  ith\  amino\ acid\ is\ in\ the\ valid\ position\end{array}\\ {}\left|E\right|,& i=n\end{array}\right. $$

where, *n* is the length of the amino acid sequence. *i* is the index of the amino acid in the sequence. *E* is the sum of the energy formed by the final folded structure. *R* is the symbolic representation of reward in this article.

#### Rigid criterion

Compared to the flexible criterion, when the agent places the next amino acid in the selection process, if this action causes the next amino acid to be placed on the lattice of the existing amino acid, the action is called invalid and needs to be re-selected until a valid action occurs. The check matrix ‘*Check*’ is introduced here. For a sequence of length *n*, the check matrix is a 2D matrix of 2*n*-1 rows and 2*n*-1 columns. The lattice position where the amino acid has been placed is marked (also called invalid position), then in this episode, this position can no longer be placed, that can be expressed as

8 $$ Check=\left\{\begin{array}{c}1,\left(p,q\right) is\ the\ invalid\ position\\ {}0,\left(p,q\right) is\ the\ valid\ position\end{array}\right. $$

where, (*p*, *q*) indicates the two-dimensional coordinates of the placement of the amino acid.

- ① Before reaching the terminal state, the reward is set to 0.
- ② When the terminal state is reached, the absolute value of the energy of the resulting structure is rewarded.

9 $$ \mathrm{That}\ \mathrm{is}\ R=\left\{\begin{array}{c}0,i\in \left(1\sim n-1\right)\\ {}\left|E\right|,i=n\end{array}\right. $$

where, *n* is the length of the amino acid sequence. *i* is the index of the amino acid in the sequence. *E* is the sum of the energy formed by the final folded structure. *R* is the symbolic representation of reward in this article.

### HP model training algorithm based on reinforcement learning with Q-learning

The algorithm for solving 2D-HP protein folding based on reinforcement learning with full states using Q-learning in rigid criterion is shown in Table 1. The program of this method is implemented on PyCharm.

**Table 1:**
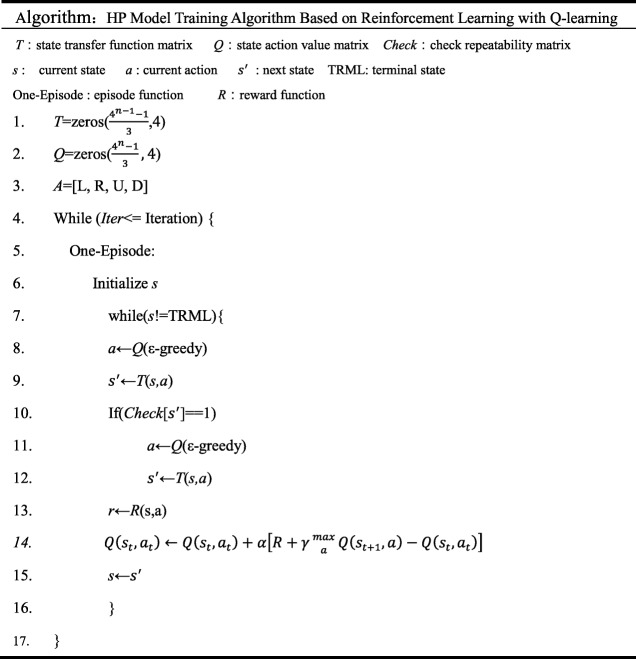
HP model training algorithm based on reinforcement learning with Q-learning

### Function approximation

The function approximation theory is an important part of the function theory. The basic problem involved is the approximate representation of the function. In reinforcement learning, for some basic methods such as dynamic programming (DP), Monte Carlo (MC) and temporal difference (TD), there is a basic premise that the state space and the action space are discrete and not too large [29]. Note that the value function of these methods is actually a table. For state value function (V), the index is the state; for state-action value function (Q), the index is a state-action pair. The process of iterative update of the value function is an iterative update of this table. If the dimension of the state space is large, or the state space is contiguous, the value function cannot be represented by a table. At this time, it is necessary to represent the value function by means of function approximation [30].

In the value function approximation method, the value function corresponds to an approximation function. From a mathematical point of view, the function approximation method can be divided into parameter and non-parametric approximation. Therefore, the reinforcement learning value function estimation can be divided into parametric and non-parametric approximation. The most commonly used is parameter approximation. When the approximation of the value function structure is determined, then the approximation of the value function is equivalent to the approximation of the parameter. The update of the value function is equivalent to the update of the parameter. In other words, it is time to use experimental data to update parameter values [31].

## Results

### Comparative experiment between rigid criterion and flexible criterion

According to two different reward settings of rigid and flexible criteria, six paper dataset sequences and ten sequences in the classic Uniref50 database are selected as experimental objects. The known information and test energy information were shown in Table 2. The parameters were set as follows: step-size parameter *α* = 0.01, exploration probability *ε* = 0.5, and learning parameter *γ* = 0.9.

**Table 2:** HP sequence set for testing

Sequence No.	HP Sequence	Length	Known lowest energy	Rigid criterion	Flexible criterion	Greedy algorithm	Partial state space
1	HPPHHPH [32]	7	−2	−2	− 2	-2	-2
2	HPHHHPHHPH [32]	10	−4	− 4	− 4	− 4	− 4
3	HPPHPPPPHPPHP [33]	13	−4	− 4	−2	− 4	−3
4	HHPHPPHPHPHHPH [32]	14	−6	− 6	− 5	−6	− 6
5	HPHHHHHHHHHPHH	14	−7	− 7	− 7	− 7	− 7
6	HHHPPHHHHHPHHH	14	− 7	− 7	−7	− 7	− 6
7	HHHHHPPHHHHPHH	14	−7	−7	−6	− 7	− 6
8	HPHHPPPHHHHHHH	14	−6	− 6	−5	− 6	− 6
9	HHHPHHPPPHHPHH	14	−6	−6	−5	− 6	−6
10	HHPHHHHHPPPPPH	14	−4	−4	− 4	− 4	− 4
11	HHPPHHHPHPPHPH	14	−6	−6	−5	− 6	− 4
12	HHHPPPPHPHHPHH	14	−5	− 5	− 5	−5	− 5
13	HPHPPHHPHPPHPHHPPHPH [34]	20	− 9	− 9	− 4	−8	− 6
14	HHHPPHPHPHPPHPHPHPPH [34]	20	−10	− 10	−7	− 9	− 8
15	HHHHHPHHPHHHHPPHHHHHH	21	− 12	− 12	−9	− 11	− 11
16	PHPPHPHHHPHPPHPHHHPPH	21	−9	− 9	−4	− 9	− 7

In Table 3, the first four sequences were chosen to compare the performance of reinforcement learning with rigid and flexible criteria. In order to avoid contingency, the rigid and flexible criteria experiments were repeated five times. The number of training iterations per round was set to 5 million, and the test was performed once every 10,000 times. In training process, the number of episodes required to converge to the lowest energy was counted as shown in Table 3.

**Table 3:** Comparison of convergence required number of sequences under two criteria (unit: / ten thousand)

Sequence No.	Criterions	1	2	3	4	5	AVG
1	Rigid	3	3	3	3	2	3
	Flexible	8	9	3	2	7	6
2	Rigid	29	28	31	9	10	21
	Flexible	37	142	43	22	41	57
3	Rigid	439	345	186	200	418	318
	Flexible	–	–	–	–	–	–
4	Rigid	238	380	256	339	114	265
	Flexible	–	–	–	–	–	–

Combination of Tables 2 and 3 showed that reinforcement learning with rigid criterion can stably find the lowest energy conformation faster than reinforcement learning with flexible criterion. For the shorter sequences (1 and 2), the number of training episodes required for agent to achieve convergence conformation by flexible criterion was greater than rigid criterion. Reinforcement learning with rigid criterion sampled an average 30,000 and 210,000 episodes to achieve the robust lowest energy conformation, which was 50 and 63% less than 60,000 and 570,000 episodes required by reinforcement learning with rigid criterion. For the longer sequences (3 and 4), reinforcement learning with flexible criterion could not find the lowest energy conformation. One possible reason was that, although flexibility criterion gave a negative reward (or penalty) for states that caused repetition, the states still had some positive Q values, and the Q values of these repeated states in rigid criterion still had an initial value of 0. Therefore, the probability of the repeated states in flexibility criterion being selected was greater than rigid criterion. And as the length of the sequence increased, the number of states that caused repetition in the full state space was also greater, and it was more difficult to find the lowest energy structure.

### Comparative experiment with greedy algorithm

Reinforcement learning with full states using rigid criterion was compared with greedy algorithm. The experimental objects were the twelve sequences in the Uniref50 data set. Similarly, in order to avoid accidentality, two methods were trained for five rounds, and the number of training iterations per round was set to 5 million, and the samples were performed once every 10,000 times. We counted the number of times the lowest energy was obtained in the last 100 samples (Table 4).

**Table 4:** The number of successfully folding to the lowest energy conformations in the last 100 episodes

Sequence No.	Methods	1	2	3	4	5	AVG
5	Full states	8	10	8	7	6	***8***
	Greedy algorithm	7	7	8	5	7	7
	Partial states	0	0	0	0	0	0
6	Full states	3	8	3	1	6	4
	Greedy algorithm	9	11	6	3	10	8
	Partial states	0	0	0	0	0	0
7	Full states	7	4	3	3	3	4
	Greedy algorithm	7	5	7	9	9	7
	Partial states	0	0	0	0	0	0
8	Full states	7	8	8	11	5	***8***
	Greedy algorithm	2	3	2	6	2	3
	Partial states	0	0	0	1	0	0
9	Full states	5	4	1	3	2	***3***
	Greedy algorithm	0	0	0	1	1	0
	Partial states	0	0	0	0	0	0
10	Full states	14	12	11	15	12	***13***
	Greedy algorithm	12	9	9	17	8	11
	Partial states	1	1	1	2	0	1
11	Full states	3	7	2	3	4	***4***
	Greedy algorithm	4	0	0	2	1	1
	Partial states	0	0	0	0	0	0
12	Full states	9	7	8	6	8	***8***
	Greedy algorithm	2	5	2	5	2	3
	Partial states	0	0	1	0	0	0
13	Full states	0	2	2	3	4	***2***
	Greedy algorithm	0	0	0	0	0	0
	Partial states	0	0	0	0	0	0
14	Full states	2	0	1	1	2	***1***
	Greedy algorithm	0	0	0	0	0	0
	Partial states	0	0	0	0	0	0
15	Full states	2	4	5	2	2	***3***
	Greedy algorithm	0	0	0	0	0	0
	Partial states	0	0	0	0	0	0
16	Full states	1	2	4	5	1	***2***
	Greedy algorithm	1	0	0	0	0	0
	Partial states	0	0	0	0	0	0

The data in bold and italic indicates the average number of successfully folding to the lowest energy conformations by the reinforcement learning with full states is more than the other two methods

It can be seen from Table 2 that reinforcement learning with full states using rigid criterion can find the lowest energy for all 16 sequences, but the greedy algorithm can only find 13 of them. From Table 4, the training process with 10 sequences was far superior to the greedy algorithm for the above 12 sequences. And the total number of times that the lowest energy was found was 300, which was greater than 205 for the greedy algorithm.

### Comparative experiment with the reinforcement learning with partial states

Reinforcement learning with full states using the rigid criterion was compared with reinforcement learning with partial states. The experimental objects and experimental settings were the same for greedy algorithm above.

In the reinforcement learning with partial states, for an HP sequence of length *n*, its state space *S* consists of 1 + 4 (n-1) states. Apart from the first amino acid that had only one state, each of the other amino acids had four different actions (up, down, left, and right) to transfer to four different states, so the number of the entire state set was expressed as 1 + 4 (n-1), so *S* = {*s*_1_, *s*_2_, …, *s*_1 + 4(n − 1)_}. For example, the state of the first amino acid is *s*_1_. In this state, the four actions of up, down, left, and right were respectively transferred to states *s*_2_, *s*_3_, *s*_4_, *s*_5_, which were all possible states of the second amino acid. On the same basis, the four actions of up, down, left and right respectively transferred to the states *s*_6_, *s*_7_, *s*_8_ and *s*_9_, which were all possible states of the third amino acid, and so on, to find all the states of the subsequent amino acids.

In Table 2, there were 8 sequences that cannot converge to the lowest energy conformations by the reinforcement learning with partial states, while reinforcement learning with full states successfully folded all sequences to the lowest energy conformations. Table 4 showed that in the last 100 episodes, reinforcement learning with full states hits the lowest energy an average five times, which was 40 and 100% higher than the three and zero times hit by the greedy algorithm and reinforcement learning with partial states, respectively. Reinforcement learning with full states achieved lower energy structures on ten out of twelve sequences than the greedy algorithm.

## Discussion

### Analysis of time complexity and space complexity

In this algorithm, for one sequence, many iterations of training are required to get its lowest energy. Therefore, the time complexity of the algorithm is determined by the length of the amino acid sequence (*N*) and the number of training iterations (*I*), that is, the time complexity is *O*(*N* × *I*). The time complexity of the ant colony algorithm for solving HP two-dimensional structure prediction is *O*(*N* × (*N* − 1) × *M* × *I*/2), where *N* is the sequence length, *I* is the number of iterations, and *M* is the number of ants. The time complexity of particle swarm optimization is *O*(*N* × *I* × *M*), where *N* is the sequence length, *I* is the number of iterations, and *M* is the number of particles. Obviously, the time complexity of the method in this paper is the smallest of the three methods, and the larger the sequence length, the more prominent the time advantage.

The space complexity is composed of state-transfer function matrix and state-action value matrix. The rows of both matrices represent states, and the columns all represent actions. The number of rows in new state-transfer function matrix is $\frac{3^{N-1}-1}{2}$ and the number of columns is 3. The number of rows in state-action value matrix is $\frac{3^N-1}{2}$ and the number of columns is 3. So the space complexity is $O\left(\frac{3^{N-1}-1}{2}\times 3+\frac{3^N-1}{2}\times 3\right)$.

### Case study

**Fig. 2:**
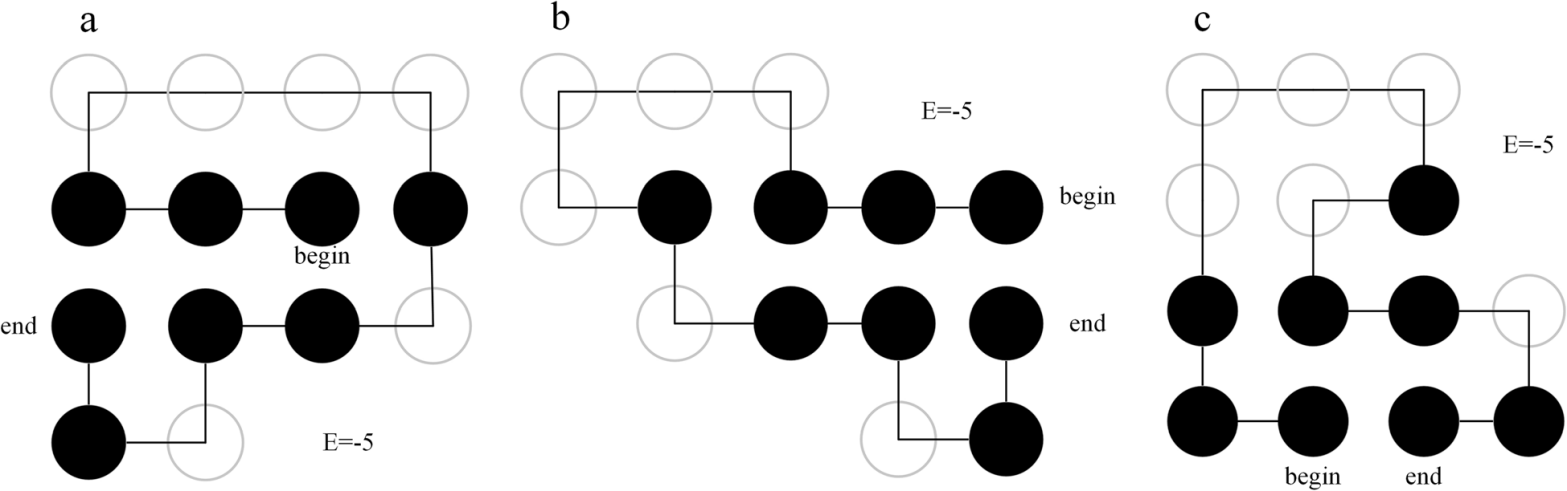
The optimal 2D conformations of sequence no.12 under the rigid criterion. **a** First optimal structure. **b** Second optimal structure. **c** Third optimal structure

**Fig. 3:**
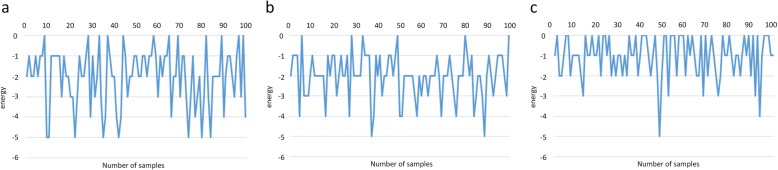
The last 100 samplings of the training process of three methods. **a** Training process sampling of full state space. **b** Training process sampling of greedy algorithm. **c** Training process sampling of partial state space

**Fig. 4:**
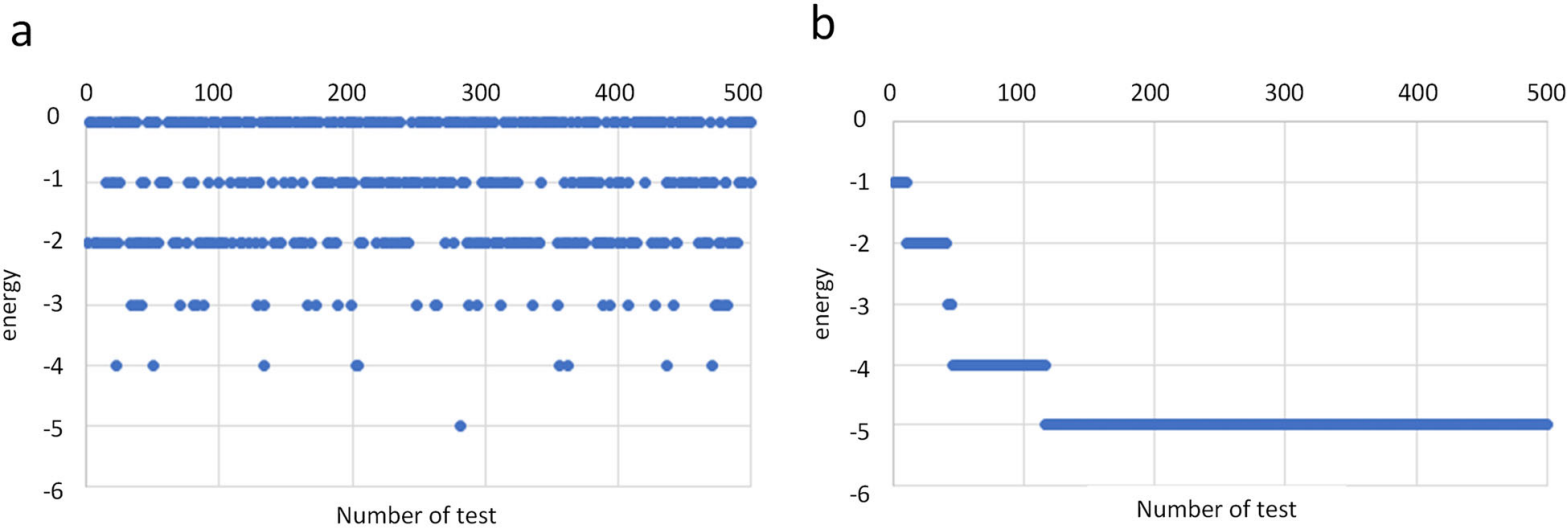
Comparison of testing process between full state space and partial state space. **a** Testing process of full state space. **b** Testing process of partial state space

Sequence 12 is a zinc finger protein 528 (fragment), which is a transcription factor with a finger-like domain and plays an important role in gene regulation. Taking sequence 12 as an example, a series of optimized structures with the lowest energy obtained by the method of this paper under rigid criterion are given, as shown in Fig. 2a-c. The results of the last 100 samples of the method and the greedy algorithm and reinforcement learning with partial states in the training exploration process are given, as shown in Fig. 3a-c. The greedy algorithm itself cannot converge, and the convergence of reinforcement learning with full and partial states in the test process is shown in Fig. 4a, b.

For reinforcement learning with full states, the agent can be trained to select the better action to obtain a lower energy structure after training for several million times, and then guarantee that the structure obtained after convergence is the optimal structure, and it can be considered that the training effect of reinforcement learning with full states is stable. However, the greedy algorithm is not ideal for training. Only several structures with the lowest energy are trained occasionally, and the accuracy of the lowest energy structure cannot be guaranteed. As a whole, reinforcement learning with full states is better than the greedy algorithm. This is because, for reinforcement learning, the agent can choose better actions based on the previous interaction with the environment during the exploration process. Therefore, as the number of training increases, the agent can select the optimal action more quickly and accurately. Also, because of the setting of the reward function, the agent is more concerned about the overall situation without being trapped in a local optimum. The calculation of each plot in the greedy algorithm is independent, and the previous experience does not help the development of the current plot. As a result, the calculation amount becomes larger and the correct structure cannot be stably obtained.

From the testing process, it can be found that reinforcement learning with full states can maintain the lowest energy and achieve stable convergence after reaching the minimum energy. In contrast, reinforcement learning with partial states has fluctuations, cannot be stably maintained, and cannot reach the convergence state. This is because each state in the full state space is uniquely determined and can only be transferred by a unique state-action pair, and the process has Markov properties. However, the state in the partial state space can be transferred by different state-action pairs, which has partial uncertainty.

### Full state space compares to partial state space

The full state space and the partial state space are two different descriptions of the state space in the 2D-HP model under reinforcement learning framework. The same point of the full and partial state spaces is that different states corresponding to each amino acid are set in advance, but they differ in the rules of the state setting. For the full state space, the number of states of subsequent amino acids is always three times the number of previous amino acid states. The state of the subsequent amino acid is obtained by a specific action of the previous amino acid in a specific state. That is to say, each state is transferred by a certain state-action pair, and the whole process has Markov properties. For the partial state space, the number of states for each amino acid except the first amino acid is four. The four states of the subsequent amino acid can be transferred from the four states of the previous amino acid through four different actions, and the whole process does not have Markov properties. The advantage of the full state space is that it can accurately find the lowest energy of the sequence and stabilize the convergence. The disadvantage is that the state space dimension is too high and the memory requirement is high, and the sequence with long length cannot be calculated. The advantage of partial state space is that the required state space is small, and it is possible to calculate a sequence with a long length. The disadvantage is that it cannot converge and cannot find the lowest energy of the sequence.

Function approximation is especially suitable for solving problems with large state space. The method described above for pre-setting the state-action value matrix and updating the state-action value matrix during the training process takes up a large amount of memory. Function approximation can be used to map the state-action value matrix to an approximation function (such as a parameter approximation function). Updating the parameter values with experimental data during the training process is equivalent to updating the state-action value, and finally a suitable approximation function is obtained. It can save memory space and solve the problem of sequence length limitation.

## Conclusion

This paper proposes a model based on reinforcement learning to solve the problem of HP model prediction. This problem is also a basic problem in computational molecular biology. The state set and state transition space are calculated according to the length of the HP sequence. The reward function is set according to different situations. The agent uses the ε-greedy strategy to select the exploration action to be rewarded, and continuously calculates and updates the Q value. Finally, the optimal solution is selected according to the converged Q-value table to obtain the optimal HP structure. In this paper, sixteen sequences were selected as experimental objects. The experimental results showed that compared with the flexible criterion, the method can converge to the optimal value function under the rigid criterion, and obtain the optimal structure of the HP model. Compared with the greedy algorithm, the algorithm can find the lowest energy more than times in the training process, highlighting the advantages of this method based on previous experience. Compared with reinforcement learning with partial states, the advantages of the stable convergence of the algorithm are highlighted. This article is a new attempt in the field of protein structure prediction with reinforcement learning, which will play an exemplary role in further three-dimensional protein structure prediction and other areas of biological information using reinforcement learning.

Besides, reinforcement learning with rigid criterion can robustly converge to the lowest energy conformations; the limitations of the method can be further improved. Firstly, although this method can be calculated for long sequences, it has higher memory requirements for computers; Secondly, in order to obtain accurate results, a large number of training events need to be taken into account, resulting in a slow convergence process and the convergence speed needs to be improved. In the follow-up study, we will further improve the forecast results from these two aspects. We believe that the reinforcement learning method applied to solve the problem of protein folding deserves further research.

## Data Availability

Dataset and source code can be access from http://eie.usts.edu.cn/prj/RLHP/index.html.

## Abbreviations

2D: Two-dimensional
2D-HP: Two-dimensional hydrophobic-polarity
AB: Hydrophobic is called A and hydrophilic is called B
ACO: Ant colony optimization
DP: Dynamic programming
EA: Evolutionary algorithm
GA: Genetic algorithm
HP: Hydrophobic polarity
MC: Monte Carlo
TD: Temporal difference
